# Design and Development of a Heterogeneous Active Assisted Living Solution for Monitoring and Following Up with Chronic Heart Failure Patients in Spain

**DOI:** 10.3390/s22228961

**Published:** 2022-11-19

**Authors:** Francisco José Melero-Muñoz, María Victoria Bueno-Delgado, Ramón Martínez-Carreras, Rafael Maestre-Ferriz, Miguel Ángel Beteta-Medina, Tomás Puebla-Martínez, Andrés Lorenzo Bleda-Tomás, Gorka Sánchez-Nanclares, Ricardo Pérez-de-Zabala, Mónica Álvarez-Leon

**Affiliations:** 1 Centro Tecnológico del Mueble y la Madera de Murcia, C/Perales s/n, 30510 Yecla, Spain; 2 Department of Information and Communication Technologies, Universidad Politécnica de Cartagena, Plaza Hospital 1, 30202 Cartagena, Spain; 3 E-Lighthouse Network Solutions S.L, C/Angel s/n, 30202 Cartagena, Spain; 4 Servicio Murciano de Salud, Edif. Habitamia 5º, 30100 Murcia, Spain; 5 MIWEnergia, Parque Científico de Murcia, 30100 Espinardo, Spain; 6 Minsait–Av. Bruselas 35, 28108 Alcobendas, Spain

**Keywords:** AAL, IoT, healthcare, prevalidation, deployment, chronic heart failure, large-scale pilot, H2020

## Abstract

Heart failure is the most common disease among elderly people, and the risk increases with age. The use of smart Internet of Things (IoT) systems for monitoring patients with chronic heart failure (CHF) in a non-intrusive manner can result in better control of the disease, improving proactive healthcare through real-time and historical patient’s data, promoting self-care in patients, reducing unneeded interaction between patients and doctors, reducing the number of hospitalizations and saving healthcare costs. This work presents an active assisted living (AAL) solution based on the IoT to provide a tele-assistance platform for CHF patients from the public health service of the region of Murcia in Spain, with formal and informal caregivers and health professionals also as key actors. In this article, we have detailed the methodology, results, and conclusions of the prevalidation phase for the set of IoT technologies to be integrated in the AAL platform, the first mandatory step before the deployment of a large-scale pilot that will lead to improving the innovation of the system from its current technology readiness level to the market. The work presented, in the framework of the H2020 Pharaon project, aims to serve as inspiration to the R&D community for the design, development, and deployment of AAL solutions based on heterogeneous IoT technologies, or similar approaches, for smart healthcare solutions in real healthcare institutions.

## 1. Introduction

In a rapidly ageing European society, there is a growing need for implementing information and communication technologies (ICT) and digital tools that improve the quality of life, independence, and overall health of older adults. In this context, the concept of ambient intelligence (AmI) appears to achieve a future where technology surrounds the users and helps them in their daily lives [1]. AmI leads to cutting-edge platforms referred to as active assisted living platforms [2,3]. The Internet of Things (IoT) [4] has also emerged as a set of technologies, systems, and design principles [5,6] that enable automation in many fields, such as remote and smart healthcare systems, playing an important role in AAL platforms and healthcare. A typical IoT environment consists of communication interfaces, sensors, advanced algorithms, and cloud interfaces [7]. Sensors are responsible for collecting data from various devices. Additionally, different communication technologies (wired and/or wireless), such as wireless sensor networks (WSN), provide network and communication infrastructure [8], while advanced algorithms are used to analyze and process data [9]. Numerous client/server requests can be exchanged in the cloud environment and allow the users to have access to various types of services simultaneously [10,11]. Due to cloud computing challenges and high requisites of emerging 5G, such as latency, reliability, resource constraints, etc. Fog computing is used to overcome these limitations and run the same applications anywhere close to users with real-time analysis and efficient decision-making features [12,13]. 

Considerable effort is being made in the R&D community to integrate IoT technologies, communications [14], databases, and computing [15], and to develop standardized and integrated AAL platforms [16], but the diversity of these types of systems has created a very fragmented market and a lack of standardized architectures and protocols [17].

In the healthcare industry, sanitary systems are being revolutionized by the IoT paradigm [18]. This plays an important role in telemonitoring in hospitals, especially at homes for elderly people with chronic diseases [19]. By using this technology, healthcare systems can experience major effects such as a reduction in response time to detect anomalies, high-quality care, low hospitalization costs, and high life expectancy [18].

In the specific case of heart failure (HF), the most common disease in elderly people and one that increases in prevalence with age [20], the use of an IoT system for monitoring patients with chronic heart failure (CHF) in a non-intrusive manner can result in better control of the disease, improving the proactive healthcare and reducing unneeded interaction between patients and doctors, thereby reducing the number of hospitalizations and saving healthcare costs. 

HF is a leading cause of hospitalization, representing 1–2% of all hospital admissions [21,22]. In Spain, the prevalence of HF is around 5% (higher than in other EU countries and USA); the rate rises with age to 8% between the ages of 65 and 74, and to 16% in persons aged 75 years and over [23]. These rates mean enormous health care resources are required, e.g., in Spain, during the 2015–2019 period, costs of HF patients were EUR 15,373 per patient, with HF hospitalizations being the most important determinant (51.0%). Medication costs represented only a small proportion of total costs [24]. The latest data published by Eurostat revealed that in 2018, HF caused 19,142 deaths in Spain, constituting 4.5% of all deaths, 3.4% in men and 5.6% in women [25]. Within this frame of reference, cardiologists and family doctors believe that the key to a better control of patients with CHF is creating a proactive healthcare solution through a non-intrusive and integrated monitoring system that unifies patients’ medical history and makes it available to all relevant healthcare professionals. This will improve the safety and efficiency of healthcare by increasing the availability of patients’ real-time information. In addition, the health and care community are convinced that it is crucial to promote self-care in patients by providing them and their caregivers with health education and training and other social and health resources.

This work presents an AAL solution based on IoT to provide a tele-assistance platform for CHF patients from the public health service of the region of Murcia in Spain. Initially focused on CHF patients aged 55 and over, the study also included formal and informal caregivers and healthcare professionals. For the three user groups, two scenarios are considered to provide a comprehensive healthcare solution:

- Angel of Health, aiming at improving health and care services and follow-up for CHF patients and involving them in the health and care process from the data perspective;
- Care@Home, aiming at reducing the dependency of CHF patients and to detect emergency situations early.

**Figure 1:**
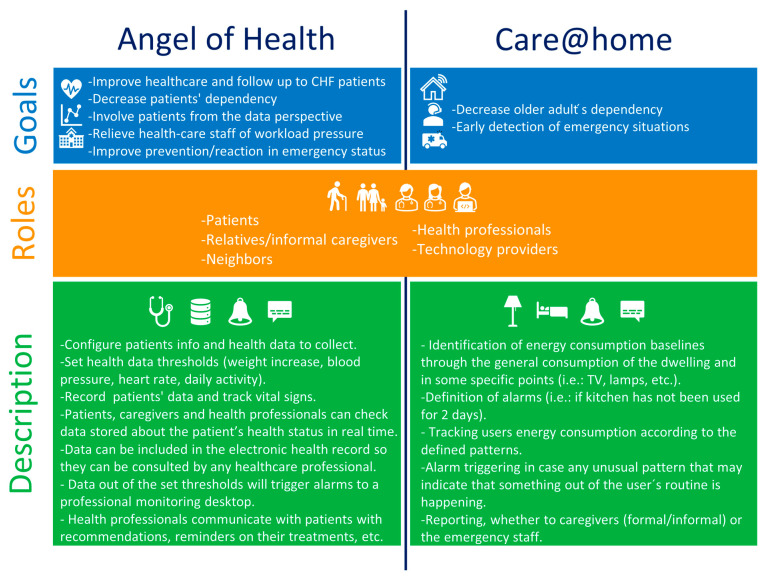
Scenarios of the Pharaon Murcia pilot.

Figure 1 summarizes the goals, roles and description of the two scenarios considered.

This work is part of the overall research in progress regarding the H2020 Pharaon Project (Pilots for Healthy and Active Ageing) [26], which aims at providing a smart and active lifestyle for Europe’s ageing population by creating a set of integrated and highly customizable interoperable open platforms with advanced services, devices, and tools in AAL, including IoT, artificial intelligence, robotics, cloud computing, smart wearables, big data, and intelligent analytics. Built upon mature existing state-of-the-art open platforms and technologies/tools, a user-centric approach is being followed for the deployment and the two-stages validation (prevalidation and large-scale pilots) in six different pilot sites: Murcia and Andalusia (Spain), Portugal, the Netherlands, Slovenia, and Italy. 

The Pharaon ecosystem integrates a big set of services and functionalities which requires the involvement of a huge number of resources in order to achieve the necessary validation and trials in real-world scenarios. The validation within Pharaon is being performed through the six large-scale pilots proposed with different types of users, requirements, and chosen functionalities. Pharaon aims to carry out the unprecedented validation of different platforms simultaneously, each supporting a wide variety of advanced and customized assistive services and tools in six different large-scale pilots with the necessary resources. For example, the Murcia pilot will validate heart failure and non-intrusive home monitoring with alarm triggering, whereas the pilot in The Netherlands focuses on community building and providing tailored advice towards user empowerment in terms of health literacy.

This approach allows a sufficiently high number of users and healthcare professionals to use the system at each pilot site for a long-term period, and an unprecedented number of use cases, services, and technologies will be tested across all pilots.

In this work, the authors present the methodology, results, and conclusions of the prevalidation phase for the set of IoT solutions and their respective AAL platforms, which will introduce the tele-assistance platform for CHF patients from the public health service of the region of Murcia. The prevalidation stage includes the participation of all actors involved: patients, formal and informal caregivers, and healthcare professionals. Their early feedback regarding the functionality and usefulness of the tested technologies and platforms is the first mandatory step before the deployment of a large-scale pilot that will lead to improving the innovation of the system from its current technology readiness level (TRL) (6) to the market (9). The goal is to provide the R&D community with inspiration in the design and deployment of AAL solutions based on heterogeneous IoT technologies, or similar approaches, for smart healthcare solutions in real healthcare institutions. 

The paper is organized as follows: Section 2 describes in detail the Pharaon Murcia pilot and the workplan followed for implementing the smart healthcare solution. Section 3 explains the engineering of user requirement tasks that were performed to identify the scenarios that define the use cases of the AAL healthcare solution and their requisites. Section 4 looks in depth at the system architecture that must perform the use cases and technical features of the hardware/software needed. Section 5 describes the roadmap implemented for the testing phase. Section 6 presents and discusses the results of the testing. Section 7 shows the conclusions and the future work recommendations. Finally, Section 8 presents a short discussion about the research limitations.

## 2. Pharaon Murcia Pilot: Overview and Workplan for the Healthcare Solution Deployment

The reduction in birth rate and the increase in life expectancy will, in the long term, lead to a progressive ageing of the population in the region of Murcia, which will manifest itself in an uninterrupted decline in the working-age population and a continued increase in the proportion of the population over 65 years of age [27]. One of the priorities of the research and innovation strategy for smart specialization in the region of Murcia [28] is related to health, biomedicine, and welfare, addressing, among other fields, housing care and ITC-supported social services, specialized care, access to services, and remote assistance. 

**Figure 2:**
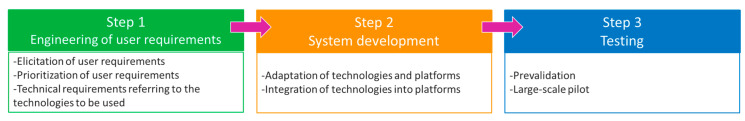
Work sequence within the Pharaon Murcia pilot.

The services and use cases to be deployed under Pharaon in the region of Murcia are aimed at building the foundations of a new telecare line in the region that will transcend the current model of health and care services that rely on the patients to notice when they need help. This new telecare model will allow patients to stay in their preferred environment and provide a more intense, effective, proactive, and less intrusive care and observation service. To do that, this work has been organized into a sequence of main steps, summarized in Figure 2. The first step consists of the elicitation and representation of the user requirements. This leads to the definition of the use case scenarios of the pilot and detailed technical requirements referring to the technologies to be used. 

The second step, informed by the results of step 1, has two main goals: to address the adaptations required for the compliance of all requirements, and the development and integration of the technologies and services in the platform. 

Finally, step 3 consists of the testing phase, organized into two stages: (1) small-scale testing with a limited number of participants representing the different target users of the Murcia–Pharaon system. They provide early feedback regarding the functionality and usefulness of the system, enabling rapid and iterative improvements to address shortcomings discovered by actual system users while its implications are still manageable. The second stage is (2) running the large-scale pilot, which involves all the pilot users in real-world scenarios for an extended length of time.

## 3. Engineering of User Requirements in the Pharaon Murcia Pilot

In [29], the authors summarized the co-design and user requirement engineering work carried out in the pilot study of the region of Murcia. During the co-design phase, the methodology for the co-design and representation of user requirements was defined as goal models with a set of components: functional goals, quality goals, and emotional goals, following work in [30]. Corresponding use case scenarios and user stories were also defined. 

The methodology entailed several up-to-date co-design methods for user requirements’ elicitation. The ISO 9241-210 standard on ergonomics of human–system interaction [31] was followed. The original plan for eliciting and representing user requirements was modified due to the COVID-19 outbreak, following three phases: 

The first phase is initial desk research on co-design workshops, data, and results from previous initiatives in which the public health service provider of the region of Murcia participated: ProEmpower [32], ReadiForHealth [33], INC3A [34], and CARPRIMUR [35]. They helped to identify an initial set of requirements from the stakeholder’s perspective in the form of functional, quality, and emotional goals.

The second phase is the design and launch of a questionnaire addressing target users of the Pharaon system that helped to define a map of barriers and opportunities in the region regarding the assistance of patients suffering from CHF. The goal was to enrich the results in the previous phase with the opinion of other representatives that were also target groups (older adults, informal caregivers, and health and care professionals). The participants were reached through an online questionnaire that was duly promoted at a regional level, and they participated in a set of virtual co-design sessions in different focus groups. In total, 250 responses (56% were patients, relatives, or caretakers, and 44% were health and care professionals) were gathered, and they helped to complete the initial goal framework. 

Next was the virtual co-design phase, consisting of the arrangement of a virtual workshop and the creation of different focus groups where representatives from the different target users of the Pharaon system were involved: health and care providers, older adults, and informal caregivers. In these workshops, different questions were posed regarding the CHF, target users, use cases, scenarios, and technologies. The discussions helped to confirm the goals and requirements identified and new ones appeared. 

**Figure 3:**
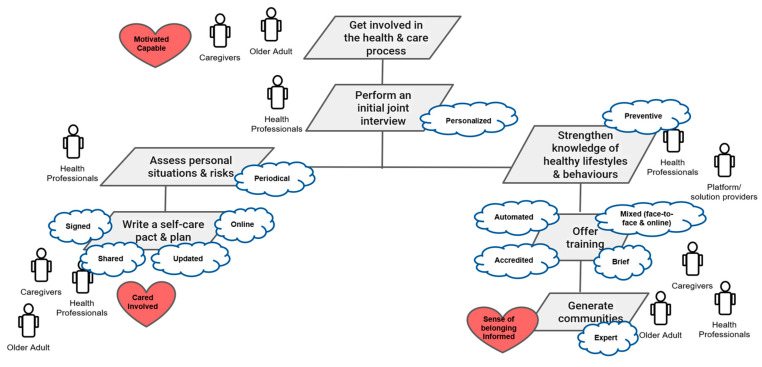
Representation of the Pharaon Murcia Pilot goal model “Get involved in the health and care process” [29].

The outcomes of the three phases resulted in the definitive set of quality, functional, and emotional goals, and this was the basis for defining the three goal models of the Pharaon Murcia Pilot (Figure 3):

- Become involved in the health and care process;
- Improve patient care;
- Detect emergency situations.

The results also helped in identifying, clarifying, and organizing the system requirements in the Murcia pilot through the definition of seven use cases and the associated technology requirements [29]:

- Become involved in the health and care process;
- Assess personal situations and risks;
- Strengthen knowledge of healthy lifestyles and behaviors;
- Improve patient care;
- Boost disease follow-up;
- Upgrade interventions;
- Detect emergency situations.

## 4. Pharaon Murcia Pilot: Architecture and System Development

**Figure 4:**
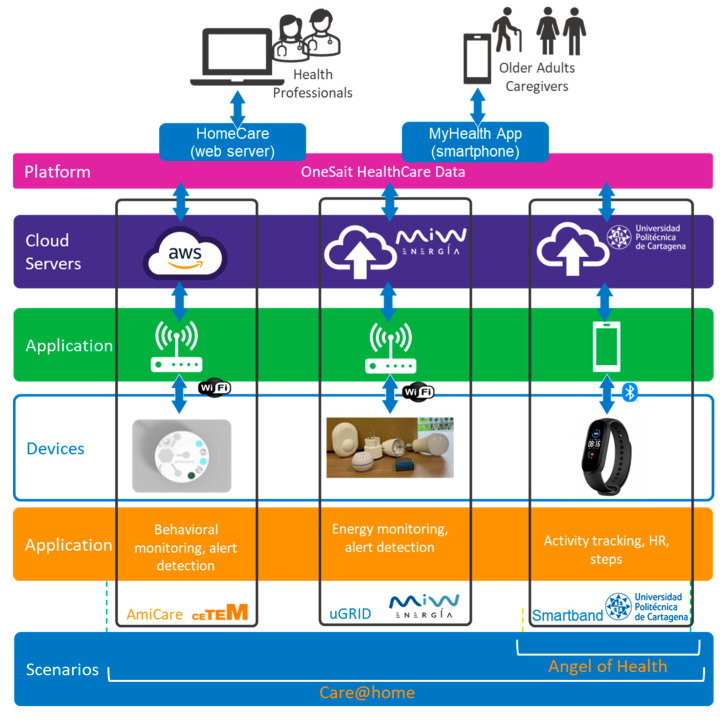
Overview of scenarios, technologies, and platform of the Pharaon Murcia pilot.

**Figure 5:**
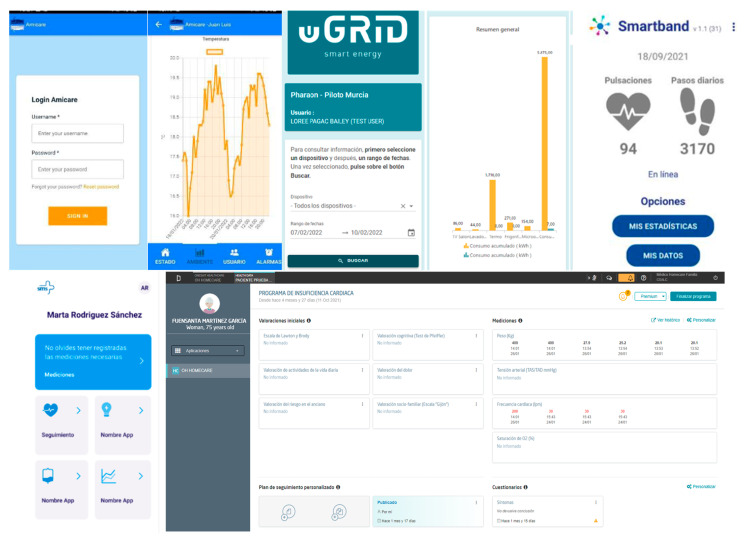
User interfaces of the technologies used in the Pharaon Murcia Pilot.

The technical requirements identified in the use cases found in step 1 of the workplan led to the selection of the technologies to be implemented (see Table 1) to cover the two scenarios, the goal models and the use cases with the software applications and platforms required (see Figure 4 and Figure 5). 

**Figure 6:**
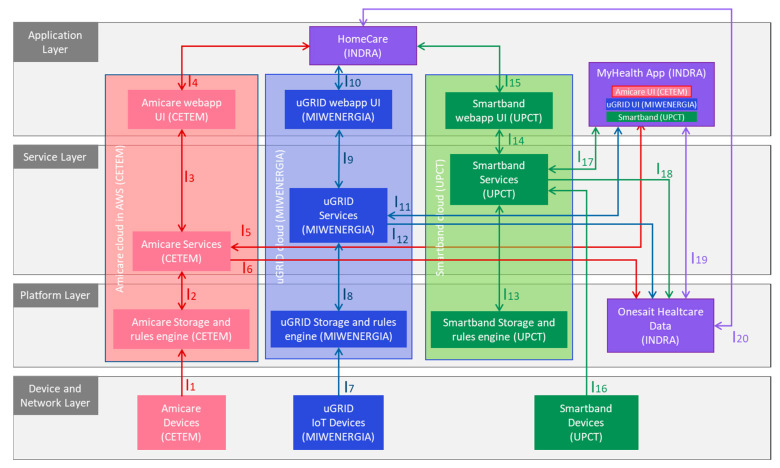
Murcia pilot high-level architecture.

During step 2, technology providers worked on the adaptations of the technologies for the compliance of all users’ needs identified in step 1 and for their integration into the Onesait Healthcare Data Platform, following the architecture agreed (see Figure 6).

**Figure 7:**
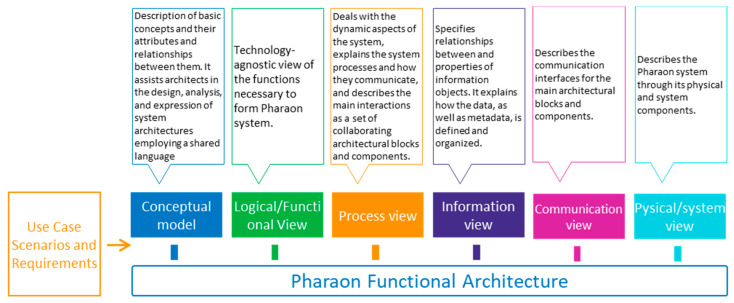
Pharaon architecture view model.

For the six pilots, the architecture description was jointly carried out as a modelling exercise, having the defined use case scenarios and requirements for each pilot as the main input. Starting from the high-level abstraction and working towards adding more details, the explicit representation of the concept and the Pharaon ecosystem becomes clearer to everyone involved. It was decided that a technology-agnostic reference architecture model should be followed, focused on standard-based, non-AAL-specific, and somewhat recognized models, along with an adaptation of the 4 + 1 view model of architecture [36] with some additional views to ensure a high degree of coherence in the process of documenting the Pharaon architecture (see Figure 7).

Based on the experience and best practice of previous similar projects and initiatives [37,38,39,40,41,42], the Pharaon Reference Architecture is built on the common approach to “horizontal” functional layering that reflects the IoT implementations across various domains, and is expanded with two additional dimensions, cross-cutting functions, and properties.

Regarding the functional layers of the Murcia pilot architecture:

1. The device and network layer that represents the functional entities used for sensing, collecting data and actuating, and enabling network connectivity and transmission of data to other higher-level functional entities, includes Amicare, Miband 5, and the power metering devices that send energy consumption data to uGRID.
2. The platform layer, responsible for integration and interoperability of basic functional components for facilitating communication amongst them and exposing the functionality of these components and databases to provide basic data storage and processing services. It comprises the storage and rule engines from Amicare, uGRID, and Smartband, and the Onesait Healthcare Data platform.
3. The service layer compiles all the services provided by Amicare, uGRID, and Smartband. It represents all functional components that support and ease application development, support mixture of different data streams, analytics and service components, and allow insights from data to be extracted and more complex data processing to be performed. Such services are executed in data centers (Smartband) or in cloud environments (Amicare and uGRID). They uniformly handle the underlying devices and networks, thus hiding the complexities of layers 1 and 2. Among these services, remote device management is included that can perform remote software upgrades, remote diagnostics or recovery, and dynamically reconfigure application processing such as setting event filters. Communication-related functions include publish/subscribe and message queue mechanisms. In general, data storage for anything from raw data to knowledge representations, and processing capabilities, such as data and event capture, filtering, and stream processing, are core services implemented by Amicare, uGrid, and Smartband.
4. The two user interfaces of the Onesait Healthcare data, the Homecare and MyHealth apps, along with the technologies’ individual dashboard integrated in MyHealth app, are included in the Application Layer, responsible for representing data in rich visuals and/or interactive dashboards and providing direct functionality of the system from a user perspective. Besides visualization, it represents other functional components that are directly responsible for enabling application-specific visualization and user interaction (such as application-specific backend services).

Figure 6 represents the architecture of the Murcia pilot at a high level, showing the elements of each of the horizontal functional layers and their cross-cutting functions, which address additional functionalities that are not linked to a single layer but whose provision requires spanning across several layers. This includes security, privacy, reliability, etc. Table 2 lists the technical description of each technology, including the interactions, technologies, protocols, and security implemented. 

## 5. Pharaon Murcia Pilot: Testing the Smart Healthcare Solution

A two-stage approach was defined for testing the Pharaon system with target users in all pilots:

1. The prevalidation stage, consisting of some initial real-world validation at a small scale, where a reduced number of participants representing the different target users (older adults, informal caregivers, and health professionals) test the technologies described above. The goal is to collect the opinion of the users regarding the use of these technologies in specific situations and to analyze the key performance indicators (KPIs) focused on the users’ level of autonomy, confidence, technology experience, usability, etc. These tests also help to detect bugs/problems/improvements and solve them before large-scale deployment.
2. The deployment of large-scale pilots with a significant number of target users testing the Pharaon system for a period of at least 12 months, where the impact is assessed at different levels according to a set of indicators agreed.

The present work includes the methods employed and results obtained during the prevalidation stage because, at the time of writing, the large-scale deployment had just started, and no significant data have been collected to evaluate the impact.

**Figure 8:**
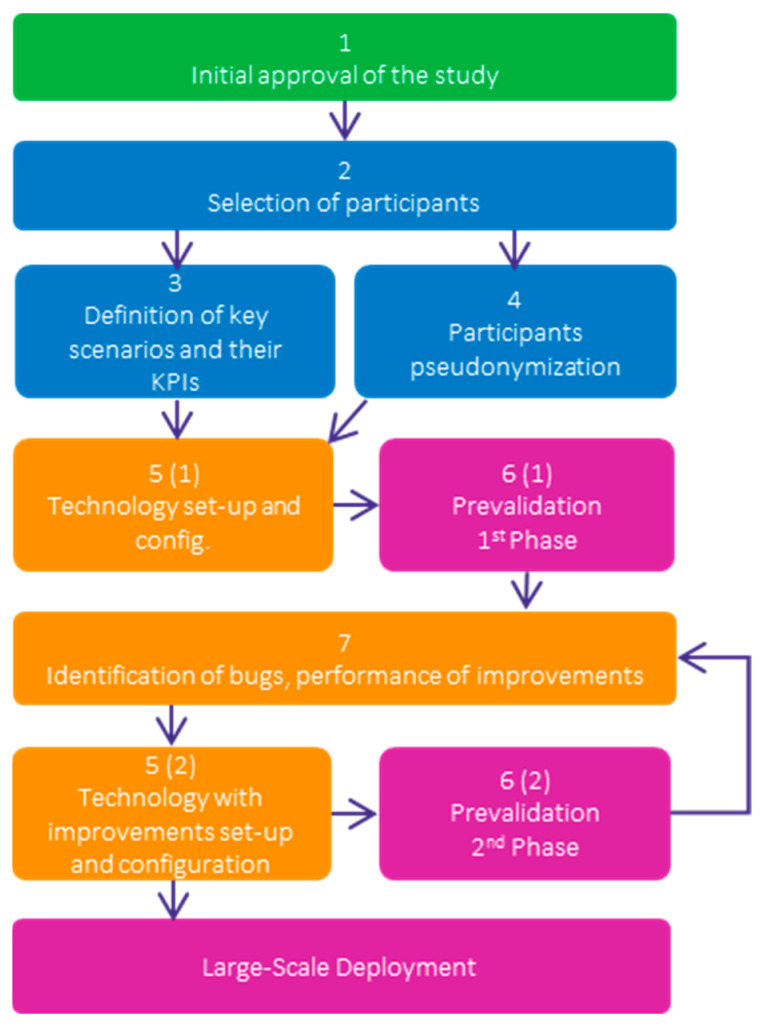
Prevalidation protocol followed by the six Pharaon pilots.

The six Pharaon pilots followed a common methodology described in a prevalidation protocol for all pilot sites to assess difficulties and willingness to use and for bug collection. For the Murcia pilot, this protocol comprised the following steps (see Figure 8):

- The initial approval of the study by the ethics authority where the prevalidation takes place. For the case of the Murcia pilot, the ethics committee of the Murcia Institute of Biomedical investigation, as the main ethics authority in the region in clinical research studies, issued a favorable opinion after the assessment of the proposed study, considering the relevance of its implementation, compliance with all basic principles in terms of ethics, and the suitability requirements of the protocol in relation to the research goals, along with the capacity of the researchers involved and the appropriateness of the available resources.
- The selection of the participants according to a set of inclusion and exclusion criteria determined by the nature and objectives of each pilot’s requirements and the living conditions, digital skills, and health status of the volunteers (see Table 3).
- The definition of the key scenarios for users to perform and assess the technologies during the test sessions (see Table 4) and the definition of the key performance indicators (KPIs) (see Table 5).
- The pseudonymization of each participant and the compilation of socio-demographic data needed for correlation calculation under impact assessment.
- Technology set-up and configuration following a predefined protocol including all safety measures, explanations to users for each device that was installed, explanation and signature of the informed consent.
- The arrangement of test sessions where participants assessed a set of predefined scenarios and KPIs in two steps as well: 6 (1) where participants assessed the technologies individually, and 6 (2) where participants assessed the platform with the technologies integrated within it.
- The identification of bugs, their registration in a specific space created in Gitlab, and further improvements.

Regarding the KPIs agreed upon (see Table 5), the one focused on usage difficulties found by the users has been evaluated through the ASQ questionnaire, with three questions (see Table 6) and a seven-level scale for each one. For the analysis of the results, the rates equal to or higher than 5 were interpreted as no difficulty. The questionnaire also comprised a “Comments” section for registering any difficulties or positive feedback about the scenario evaluated. The KPI focused on willingness to use was assessed by the System Usability Scale (SUS) questionnaire (see Table 7), a method that allows us to obtain a general view of subjective assessments of usability for a wide variety of products and services, including hardware, software, mobile devices, websites, and applications. Due to the heterogeneity of ICT solutions that comprise the Pharaon pilots, SUS was the user-testing tool agreed upon by all partners in the Pharaon project, even though there is other research focused on healthcare solutions that use the popular Technology Acquisition Model (TAM) questionnaire as the tool to measure the technology acceptability [44]. 

The SUS scale comprises 10 items that participants had to score from 1 to 5 once the scenarios of each technology were finalized; the calculation of the results based on the participants’ scores gives a general score between 0 and 100.

SUS Score = 2.5 × [(SUS1 + SUS3 + SUS5 + SUS7 + SUS9-5) + (25 − SUS2 − SUS4 − SUS6 − SUS8 − SUS10)]

Table 8 shows the general guideline on SUS Score interpretation provided by some authors [45]. The prevalidation protocol considered those solutions as accepted if the average score of SUS was graded as A (excellent) or B (Good).

Finally, it is necessary to provide a deeper explanation of the way the steps 5–7 were performed during the prevalidation (see Figure 8):

- Steps 5 (1) and 6 (1) were carried out together for each participant (patient and his/her caregiver), that is, on a fixed date, the technologies were installed and configured at the participant’s home. In that visit, the patient and his/her caregiver took time testing the technologies individually, performing the predefined scenarios for each technology shown in Table 4 and filling out the ASQ and SUS questionnaires.
- Step 7 was carried out, analyzing the results gathered in the steps before, identifying and solving bugs and problems, and improving the technologies accordingly. In some cases, the technologies or their improvements could involve new set-ups or configurations; then, step 5 (2) was included.
- After the first 5-6-7 iteration, step 6 (2) was performed in different sessions, organized by type of participant. On the one hand, healthcare professionals participated in the testing sessions, performing the scenarios defined with the Onesait Homecare Healthcare software tool, where all technologies were integrated (see Table 2). After that, they completed the ASQ and SUS. On the other hand, patients and caregivers performed the second testing round with the improved technology result of step 7 and 5 (2), and all of them already integrated in the MyHealth App. As in the previous testing session, they completed the ASQ and SUS.

It is important to remark that the second prevalidation phase was affected by COVID-19 restrictions, and some testing sessions were performed in hybrid mode following the premises of the Murcia health service.

## 6. Results 

During the prevalidation stages, 44 people participated in testing sessions 6 (1) and 6 (2), namely:

- Prevalidation phase 1. Testing sessions in 6 (1): nine sessions were performed with a total of fifteen participants—nine older adults and six informal caregivers—from four municipalities of the region (Murcia, Cartagena, Alcantarilla and Yecla).
- Prevalidation phase 2. Testing session in 6 (2): twelve sessions were performed with 14 health professionals, ranging from cardiologists, to nurses, family doctors, neurologists, and pharmacists, and two sessions with eight older adults (two of them online), and seven informal caregivers.

Table 9 shows data from participants related to the following five variables: type of user, gender, year of birth, digital skills, and education level.

Note that, although the number of participants (samples) in the prevalidation phase may seem small, many practitioners in the industry have adopted the five-users rule as standard practice for user-testing, which points out that five participants are enough for getting a useful result for testing usability. 

All participants filled in the ASQ and SUS. The statistical methodology used consisted of a set of sequential procedures for handling the qualitative and quantitative research data: collection, counting, presentation, synthesis, and analysis. The analysis was performed using descriptive and exploratory analysis. The descriptive analysis served to describe the set of data, thus obtaining the parameters that distinguish the characteristics of a set of data. The reasons for carrying out this analysis are that it allowed us to know, in detail, the information we had and to know the way in which the information was structured. It helps to make deductions directly from the data and parameters obtained. The exploratory analysis consisted of a set of statistical techniques whose purpose was to obtain a basic understanding of the data, allowing the detection of salient features, such as unexpected and outliers. In this work, the mean was the main statistical technique used. The mean scores and global mean for each questionnaire and question are summarized in Table 10, Table 11, Table 12 and Table 13. 

In the first prevalidation phase, the mean score of the ASQ questions for each technology/scenario resulted in a value higher than 5 for all questions (see Table 10). Only one of nine patients (11.11%) scored questions ASQ1 and ASQ2 below 5 for Amicare scenario 1, considering the scenario somewhat difficult and not so fast to complete. The global mean for all questions reveals that almost all participants performed the requested scenarios with no difficulties, in an acceptable time, and felt supported throughout the process.

Regarding usability, the mean SUS scores obtained from all technologies (Table 10) were above 80.3, achieving an “A” grade (Excellent). Only two participants rated the smartband solution with 80 points. 

From the results of the prevalidation phase 1, we concluded that the KPIs were reached for all technologies/scenarios, fulfilling the requisites of the first testing phase of prevalidation. 

In the second prevalidation phase, the mean scores (Table 12 and Table 13) were better than in the previous sessions in almost all technologies/scenarios, which means that participants are more satisfied with the solution in terms of difficulty and usability. From the eight patients that tested the MyHealth App module from Onesait Healthcare Data, the rates given to the SUS questions of only one patient (12.5%) achieved a score below 80.5. The six caregivers scored all individual ASQ questions with 5 or above, and for the Homecare module from Onesait Healthcare Data, at least one ASQ question from scenarios 5, 6, 9, 10, and 12 were scored as 4 by four professionals (28.5%). The rates given to the SUS questions of two health professionals (12.28%) received a score below 80.5.

Finally, it is important to mention that, during the first prevalidation phase, only two bugs (minor issues) were reported in Gitlab related to the smartband solution that were solved and the software was updated for step 6 (2). No bugs were reported during the second phase of pre-validation.

## 7. Conclusions and Future work

This work presented an innovative AAL solution built upon novel and mature existing IoT technologies with the aim of providing a tele-assistance platform for CHF patients from the public health service of the region of Murcia in Spain. The solution has been designed with a user-centric approach, but also involves formal and informal caregivers and health professionals. From the authors’ point of view, this work has been used as a useful guideline to provide the R&D community with inspiration for the design and deployment of AAL solutions based on heterogeneous IoT technologies, or similar approaches, for smart healthcare solutions in real healthcare institutions.

It is important to note some key points of the work presented, as follows. The starting point must be a set of needs identified. Normally, healthcare professionals or institutions are the ones who detect the need. In this work, the needs were detected by the healthcare institution of the Murcia region for CHF patients. After that, a workplan of well-defined steps must be agreed upon by all parties. It must include at least engineering of user requirements, design of the system architecture and development, and testing of the solution for a final step of large-scale deployment. For each step, a clear methodology must be defined and executed with a set of expected outputs identified, which will serve as input for the next step(s). The results obtained from each step must be analyzed, identifying the improvements to implement and lessons learnt. 

Regarding the steps defined for this work in depth, it is important to highlight some important issues regarding timing, participants, key points of the execution, and lessons learnt. In the engineering of user requirements, at least six months of implementation were required, with the participation of representatives of all target users involved in the final AAL solution. In a healthcare solution such as the one presented, patients, formal and informal caregivers, and healthcare professionals are expected to be involved at least. Moreover, a high number of participants help to better define the co-design and representation of user requirements as goal models, use case scenarios, and user stories. In this work, up to 250 people participated in the different initiatives, workshops, and meetings organized to define them.

The step focused on architecture definition, development, and integration is a technical approach with a high load of software development. The duration will depend on the maturity of the technologies involved, the functional requirements, and the final healthcare product expected. In this work, this step was planned for one year of work and was focused on two sequential goals. First, the definition of a functional architecture with a set of functional layers and others spanned across several layers, such as security, privacy and reliability, defining the interactions and protocols to use. Second, the development and adaptations of three innovative IoT technologies—Amicare, Smartband, uGRID—and the Homecare and MyHealth App as modules of the Onesait Healthcare Data platform. The main lesson learnt was the importance of using open and interoperable interfaces. Although some IoT solutions are shown as plug and play, minor or major adoptions must almost always be carried out. Data privacy and security are also a must. 

Regarding the prevalidation step of the AAL solution, the mandatory assessment of the functionality, usability, and acceptance of the solution by the final users is noteworthy. It entails the participation of all actors involved in this work: patients, formal and informal caregivers, and health professionals. Moreover, all technologies must be monitored during the prevalidation to ensure good performance. In this work, the prevalidation involved the testing of each stand-alone IoT technology and the testing of all IoT technologies integrated in the final healthcare platform solution. Over two months, the authors collected feedback on the tested solution in both phases with 44 participants involved. From the results, the authors concluded that, on average, the rate of acceptance and usability of all technologies and software solutions was higher than expected, and no major bugs or unexpected issues were detected. Then, the product will be ready to be launched in a large-scale pilot.

As a final conclusion, the steps executed, explained in detail in this paper, have been essential in utilizing the future actions that will improve the innovation of the healthcare platform solution presented at its current TRL of 6, to launch a final development in view of creating a large-scale pilot, where around 450 participants are expected in the Murcia region, and to design and develop the impact, exploitation, and business plan. 

## 8. Discussion

Although the work presented has been performed to a high professional quality, following guidelines agreed under a H2020 European project framework, and the results of the work performed have been very positive and useful, it is also important to mention that the authors have identified in this contribution a set of limitations without undermining the quality and integrity of the research. In this section, we summarize them and discuss how they could impact the work and results, and we provide countermeasures and alternatives for future work if this applies. 

This work has been performed thanks to the collaborative work of six different organizations and professionals of different disciplines. In addition, more than 300 participants have been reached to participate during the different stages of the research work, which involved difficult organization and coordination. The COVID-19 outbreak added more difficulties due to the impossibility of performing face-to-face meetings and other organizational problems, which resulted in all tasks being redesigned and rescheduled. Time constraints also affected the research negatively, setting as future work some tasks that could have been finished or that at least had sufficient results which could have been included at the time this contribution was written, e.g., the experience of the large-scale pilot under execution. 

Some research/development limitations have been also identified during the development of the integrated ICT solution, due to the architecture complexity and high-quality requirements and/or the lack of knowledge of some specific programming languages mandatory for the final integration with the platforms provided by Indra-Minsait. 

Data and statistics have also been a limiting factor of the research performed. During the prevalidation phase, 44 participants were reached. Although it is a sufficient sample size for analyzing usability using SUS, the use of other evaluation questionnaires may need more samples, or some researchers may even consider 44 a low number of participants in a testing phase of a large healthcare ICT solution. 

The lack of similar research works in the scientific literature has also limited the comparison with similar approaches and the enrichment that a state of art offer to the research contributions. 

Finally, we remark that the work developed has been an ad hoc solution for specific target users and healthcare providers, and may show a strong regional focus, limiting its impact. However, some regional, national, and international stakeholders have already shown interest in the work for replicating the solution, or part of it, for other healthcare systems or types of patients (e.g., palliative healthcare). 

## Figures and Tables

**Table 1:** Technologies of the Pharaon Murcia pilot.

Technology Name	Technology Classification	Description	Murcia Pilot Scenario	Hardware/Software Components	Technical Pre-Requirements
Amicare (Technical Research Centre of Furniture and Wood of the Region of Murcia)	Indoor non-intrusive tracking of daily habits with configurable triggering alarms on caregivers’ smartphone	Non-invasive system that monitors older adults’ daily habits and contributes to the peace of mind of relatives and caregivers thanks to the power of IoT. Out-of-sight textile, movement, and ambient sensors are safely connected through the cloud to any smartphone app with a preregistered user. The app can be configured and personalized by its user to trigger alarms if certain actions happen (or do not happen) within specified time windows, such as: “if not in bed at any time between 22:00–0:00”, “if on the coach for more than 3 consecutive hours”, “if away from bed for more than 45 consecutive minutes between 0:00 and 6:00”.	Care@Home	Hardware components: -Textile sensor pad; -HW box including processor, wireless communications, on-board sensors (movement, humidity, luminosity, temperature), and textile sensor connectivity; -App for Android device (smartphone or tablet); -Web-based user interface for group of users (e.g., nursing homes).	Wi-Fi connection is required
uGRID (MIWenergía)	Energy Management Platform	The uGRID software aims to digitize energy consumption, providing the final consumer with more information about their demand of electrical energy. The purpose is to achieve the maximum possible energy efficiency and to control the electricity consumption by setting alerts and generating reports.	Care@Home	Hardware Components: -Dedicated database server; -Dedicated platform web server; -Power metering devices. Software Components: -Web platform based on PHP/Javascript; -MariaDB database (MySQL).	Wi-Fi connection is required
Smartband Solution (based on Mi band 5 of Xiaomi) (Universidad Politécnica de Cartagena)	Wearable	Through a commercial smartband wirelessly connected to the patient’s smartphone, the smartband solution allows recording, in a non-invasive way, real-time (and historical) data of patients regarding their heart rate and daily activity in a number of steps. The novelty of this solution is that it also provides the patient data to his/her caregiver and to his/her healthcare professional. Moreover, the healthcare professional can configure alarms if heart rate is lower or higher than a certain threshold.	Care@Home Angel of Health	Hardware Components: -Smartband; -Charger. Software Components: -App for Android devices (smartphone or tablet) to track user’s heart rate and daily activity, reporting real-time and historical data to patients, caregivers, and healthcare professionals.	-Connection to a smartphone through Bluetooth is required -Internet connection is required for the smartphone
Onesait Healthcare Data (Indra Minsait)	Telemedicine Health Platform	It allows the treatment and follow-up of chronic patients at home. Through its two user interfaces, the system integrates the above technologies and offers tools for the bidirectional communication among healthcare professionals in the clinical setting and patients at home, so that patients are provided with personalized treatments according to their clinical conditions and progress. Onesait Healthcare currently allows the remote monitoring of patients suffering from specific chronic diseases, such as CHF, diabetes, or hypertension, while enabling mobility-ubiquity of users, personalization, and interoperability between systems (seamless systems).	Care@Home Angel of Health	Software Components: -MDM: includes managers for population information, catalogues and resources, along with a single sign on and an Audit and Log Server; -Onesait Healthcare Global Repository: health data storage standardized under HL7 FHIR^®^ focused on interoperability; -Onesait Healthcare Professional Desktop: for professionals, citizens, managers, researchers, etc. that allows access to the different tools or applications; -Onesait Healthcare HomeCare: for health professionals to monitor patients; -Form Builder: tool for the configuration and parameterization of health questionnaires; -Alert: in charge of managing alarm triggers and its visualization by the operators to whom they are addressed; -MyHealth App: supports the functionalities to be used by Older Adults and their Caregivers.	-Access to smartphone is required for Patients and Caregivers. -Access to personal computer is required for health professional.

**Table 2:** Interactions between the elements of the Murcia pilot architecture and their technologies, protocols, and security.

	Interaction	Technologies and Protocols	Security
Amicare 1–6	I1	MQTT over Wi-Fi	Authentication
	I2	VNP	Authentication
	I3	REST API	TLS, AWS STS (Security Token Service)
	I4	HTTPS	JWT, SSL
	I5	REST API	TLS, AWS STS (Security Token Service)
	I6	REST API	Authentication in Rest services is through a JWT token signed with its private key
uGRID 7–12	I7	MQTT over Wi-Fi	Login
	I8	REST API	Uses SSL, API Key
	I9	REST API	Uses SSL, API Key
	I10	.NET Core (Blazor Server)	Uses SSL, Login
	I11	Ionic + Angular + Cordova	Uses SSL, Login
	I12	API REST/REST FHIR	Authentication in Rest services is through a JWT token signed with its private key
Smartband 13–18	I13	Local host socket	Authentication
	I14	HTTPS	Authentication (SSL)
	I15	HTTPS	JWT, SSL
	I16	Bluetooth	Authentication
	I17	HTTPS REST API	JWT, SSL
	I18	API REST/REST FHIR	Authentication in Rest services is through a JWT token signed with its private key
Onesait Healthcare Data 19–20	I19	API REST/REST FHIR	Oauth 2
	I20	API REST/REST FHIR	Oauth 2

**Table 3:** Inclusion and exclusion criteria defined for users involved in the Pharaon Murcia pilot.

User Type	Inclusion Criteria	Exclusion Criteria
Older Adults	- CHF with any level of left ventricular ejection fraction (LVEF); - Frailty score preferably between 4 (vulnerable) and 6 (moderately frail) ^1^; - Signed consent form; - Digital skills; - Availability of certain electronic devices and Wi-Fi Connection; - Digital skills, knowledge in e-health devices or higher knowledge in his/her pathologies.	- Planned heart transplantation, cardiac surgery, or left ventricular assisted device (LVAD) implant; - Chronic renal replacement therapy (haemodialysis, peritoneal dialysis, or transplant); - Evidence of active or suspected cancer or a history of malignancy in the last five years; - Inability or unwillingness to provide informed consent or to comply with study requirements; - Life expectancy below 1 year (due to another cause excluding HF) or a frailty scale of 7 and above; - Serious psychiatric illness; - Participation in another clinical trial.
Caregivers	- Older than 18 years old; - Digital skills; - Availability of electronic devices.	None
Health Professionals	- Digital skills; - Proactivity; - Motivation.	None

^1^ According to the clinical frailty scale of the Canadian Study on Health and Ageing (CSHA) living at home [43].

**Table 4:** Scenarios defined for each technology of the Pharaon Murcia pilot.

Technology	User Type	Scenario
Amicare	Patient	You want to find out the time you went to bed on a specific date according to Amicare. You want to know the humidity in your home today.
	Caregiver	You want to check that Mrs. X’s home temperature is between 19 °C and 25 °C. You want to see if Mrs. X’s movements in her flat were regular over the last 3 days. You want to check that you will receive an alert when the alarm configuration is fulfilled.
uGRID	Patient	You want to know your real-time electricity consumption. You want to know if the kitchen has been used for cooking. You want to know which electrical device consumes the most electricity in your home.
	Caregiver	You want to know which electrical device consumes the most electricity at the patient’s home. You want to know if Mrs. X has used the washing machine this week. You want to see if you have received any alarms or events about changes in the power consumption patterns of Mrs. X.
Smartband	Patient	You want to see your heart rate. You want to know how many steps you have taken today.
	Caregiver	You want to find Mr. X´s heart rate. You want to know how many steps Mr. X has taken today.
Onesait Healthcare Data	Patient	You want to manually register a weight value/blood pressure. You want to access your CHF program to fill in a health questionnaire. You want to access your CHF follow-up program and consult your personal care plan.
	Caregiver	You want to manually register a weight/blood pressure value of Mrs. Z. You want to access to the follow-up details of Mrs. Z. You want to access Mrs. X´s CHF program to fill in a health questionnaire. You want to access Mrs. X´s CHF follow-up program and consult her personal care plan.
	Health Professional	You want to access Mrs. X’s CHF follow-up program. You want to perform an initial clinical assessment of Mrs. X. You want to create and publish the personal care plan (PCP) for Mrs. X through a template so she can consult it through the MyHealth App. You want to customize what measures (vital signs) Mrs. X must include in the CHF follow-up. You want to include a weight range between 50 and 62 kg, outside of which you will receive an alert for Mrs. X. You want to customize what health questionnaires Mrs. X must fill in from MyHealth App and how she will have them available: only once, or any time to be filled whenever she wants. You want to consult the most recent records of Mrs. X’s heart rate and her heart rate history (Smartband). You want to consult the history of personalized follow-up plans of Mrs. X (the details of the plan, which professional published it and when). You want to know if Mrs. X has used the washing machine (or any other device that is being monitored) this week (MIW+). You want to check that you will receive an alert when the alarm configuration is fulfilled (Amicare). You want to check what patients have alerts from the list of patients of the CHF follow-up program. You want to finalize Mrs. X´s CHF follow-up program.

**Table 5:** Key performance indicators used during the prevalidation stage.

KPI	Evaluation Area	Data Source	Kind of Data Source	Target
Identification of potential bugs	Number of bugs detected during test session	Gitlab Platform	Test of functionalities and usage scenarios	Table of bugs number
Usage difficulties	User acceptance	Questionnaire	After-Scenario Questionnaire (ASQ)	Medium score ≥ 5
Willingness to use	User acceptance	Questionnaire	System Usability Scale (SUS)	Medium score ≥ 68

**Table 6:** Sample questions in the After Scenario Questionnaire (ASQ).

**ASQ1: Overall, I Am Satisfied with the Ease of Completing the Tasks in This Scenario**.										
Disagree	1	2	3	4	5	6	7	Agree		
	Comment: specification of agreement or disagreement.									
**ASQ2:** **Overall, I Am Satisfied with the Amount of Time It Took to Complete the Tasks in This Scenario.**										
Disagree	1	2	3	4	5	6	7	Agree		
	Comment: specification of agreement or disagreement.									
**ASQ3:** **Overall, I Am Satisfied with the Support Information (Online-Help, Messages, Documentation) When Completing the Tasks.**										
Disagree	1	2	3	4	5	6	7	Agree		
	Comment: specification of agreement or disagreement.									

**Table 7:** System Usability Scale (SUS).

**SUS1.** **I Think That I Would Like to Use This System Frequently.**						
Strongly disagree	1	2	3	4	5	Strongly agree
**SUS2.** **I Found the System Unnecessarily Complex.**						
Strongly disagree	1	2	3	4	5	Strongly agree
**SUS3.** **I Thought the System Was Easy to Use.**						
Strongly disagree	1	2	3	4	5	Strongly agree
**SUS4.** **I Think I Would Need the Support of a Technical Person to Be Able to Use This System.**						
Strongly disagree	1	2	3	4	5	Strongly agree
**SUS5.** **I Found the Various Functions in This System Were Well Integrated.**						
Strongly disagree	1	2	3	4	5	Strongly agree
**SUS6.** **I Thought There Was Too Much Inconsistency in This System.**						
Strongly disagree	1	2	3	4	5	Strongly agree
**SUS7.** **I Would Imagine That Most People Would Learn to Use This System Very Quickly.**						
Strongly disagree	1	2	3	4	5	Strongly agree
**SUS8.** **I Found the System Very Cumbersome to Use.**						
Strongly disagree	1	2	3	4	5	Strongly agree
**SUS9.** **I Felt Very Confident Using the System.**						
Strongly disagree	1	2	3	4	5	Strongly agree
**SUS10.** **I Needed to Learn a Lot of Things Before I Could Get Going with This System.**						
Strongly disagree	1	2	3	4	5	Strongly agree

**Table 8:** General guideline of SUS Score interpretation [45].

SUS Score	Grade	Adjective Rating
>80.3	A	Excellent
68.0–80.3	B	Good
68	C	Okay
51–68	D	Poor
<51	E	Awful

**Table 9:** Socio-demographic information of the recruited participants.

Type of User	Gender	Year of Birth	Digital Skills (*)	Education Level (**)	Pre-Validation Phases Involved
Patients	Female	1964	Some Experience	EQF Level 6	Phase1, Phase 2
	Female	1946	Some Experience	EQF Level 7	Phase1, Phase 2
	Male	1959	Some Experience	EQF Level 6	Phase1, Phase 2
	Male	1936	Some Experience	EQF Level 6	Phase1, Phase 2
	Male	1968	Experienced/Proficient	EQF Level 7	Phase1, Phase 2
	Female	1946	Some Experience	EQF Level 6	Phase1, Phase 2
	Male	1955	Some Experience	EQF Level 4	Phase1, Phase 2
	Female	1957	Some Experience	EQF Level 6	Phase1, Phase 2
	Female	1962	Some experience	EQF Level 5	Phase1, Phase 2
Caregivers	Female	1962	Some experience	EQF Level 5	Phase1, Phase 2
	Male	1995	Experienced/Proficient	EQF Level 7	Phase1, Phase 2
	Female	1972	Some experience with autonomy	EQF Level 5	Phase 2
	Male	1945	Some experience	EQF Level 6	Phase1, Phase 2
	Female	1956	Experienced/Proficient	EQF Level 3	Phase1, Phase 2
	Female	1955	Some experience	EQF Level 5	Phase1, Phase 2
	Male	1955	Some experience with autonomy	EQF Level 7	Phase1, Phase 2
Health Professionals	Male	1970	Some experience with autonomy	EQF Level 8	Phase 2
	Male	1977	Some experience with autonomy	EQF Level 8	Phase 2
	Female	1962	Some experience with autonomy	EQF Level 6	Phase 2
	Female	1982	Some experience with autonomy	EQF Level 6	Phase 2
	Male	1965	Some experience with autonomy	EQF Level 8	Phase 2
	Female	1974	Some experience with autonomy	EQF Level 6	Phase 2
	Male	1969	Experienced/Proficient	EQF Level 7	Phase 2
	Male	1974	Some experience with autonomy	EQF Level 8	Phase 2
	Female	1981	Some experience with autonomy	EQF Level 8	Phase 2
	Female	1973	Some experience with autonomy	EQF Level 8	Phase 2
	Female	1959	Some experience with autonomy	EQF Level 6	Phase 2
	Male	1973	Some experience with autonomy	EQF Level 8	Phase 2
	Female	1960	Some experience with autonomy	EQF Level 8	Phase 2
	Male	1979	Some experience with autonomy	EQF Level 8	Phase 2

(*) No experience, Some experience, Some experience with autonomy, Experienced/Proficient. (**) EQF Level 1: Primary Education; EQF Level 2: Academic Secondary School Lower Cycle, New Secondary School, and Lower Secondary School; EQF Level 3: Academic Secondary School Upper Cycle, Intermediate and Higher VET (up to 3rd grade); EQF Level 4: Post-secondary non-tertiary education; EQF Level 5: Short-cycle tertiary education; EQF Level 6: Bachelor’s Degree, Higher Apprenticeship; EQF Level 7: Master’s Degree, postgraduate certificate and diplomas; EQF Level 8: Doctorate or Equivalent.

**Table 10:** ASQ mean scores for the individual technologies during the pre-validation phase 1.

Technology	Type of User	Scenario	Mean Scores for Individual Questions			Global Mean
			ASQ1	ASQ2	ASQ3	
Amicare	Patients	1. You want to find out the time you went to bed on a specific date according to Amicare.	6.00	5.89	6.56	6.15
		2. You want to know the humidity in your home today.	6.89	6.78	6.67	6.78
	Caregivers	1. You want to check that Mrs. X’s home temperature is between 19 °C and 25 °C.	6.67	6.83	7.00	6.83
		2. You want to see if Mrs. X’s movements in her flat were regular over the last 3 days.	6.00	6.17	6.50	6.22
		3. You want to check that you receive an alert when the alarm configuration is fulfilled.	6.33	6.50	6.50	6.44
uGRID	Patients	1. You want to know your real-time electricity consumption.	5.75	5.38	6.25	5.79
		2. You want to know if the kitchen has been used for cooking.	5.88	5.88	6.00	5.92
		3. You want to know which electrical device consumes the most electricity in your home.	6.25	6.25	6.38	6.29
	Caregivers	1. You want to know which electrical device consumes the most electricity at the patient´s home.	6.67	6.83	7.00	6.83
		2. You want to know if Mrs. X has used the washing machine this week.	6.00	6.17	6.50	6.22
		3. You want to see if you have received any alarms or events about changes in power consumption patterns of Mrs. X.	6.33	6.50	6.50	6.44
Smartband Solution	Patients	1. You want to consult your heart rate.	6.86	6.86	6.71	6.81
		2. You want to know how many steps you have taken today.	7.00	7.00	6.86	6.95
	Caregivers	1. You want to consult Mr. X´s heart rate.	5.33	5.50	5.67	5.50
		2. You want to know how many steps Mr. X has taken today.	5.67	5.50	5.83	5.67

**Table 11:** SUS mean scores for the individual technologies during the pre-validation phase 1.

Technology	Type of User	Mean Scores for Individual Questions										SUS Mean Score
		SUS1	SUS2	SUS3	SUS4	SUS5	SUS6	SUS7	SUS8	SUS9	SUS10	
Amicare	Patient	4.44	1.44	4.78	1.78	4.67	1.33	4.44	1.44	4.56	1.44	88.61
	Caregiver	4.67	1.83	4.50	1.33	4.67	1.83	3.33	1.33	4.50	1.50	84.58
uGRID	Patient	4.00	1.50	4.38	1.75	4.38	1.63	3.88	1.50	4.50	1.75	82.50
	Caregiver	4.50	1.33	4.83	1.33	4.17	1.33	4.17	1.17	4.83	1.00	90.83
Smartband Solution	Patient	4.86	1.14	4.86	1.43	4.71	1.14	4.86	1.14	4.29	1.29	93.57
	Caregiver	4.60	2.00	4.80	1.20	5.00	1.00	3.80	1.20	4.80	1.20	91.00

**Table 12:** ASQ mean scores for Onesait Healthcare Data with the integrated technologies during the pre-validation phase 2.

**Technology**	**Type of User**	**Scenario**	**Mean Scores for Individual Questions**			**Total Mean**
			**ASQ1**	**ASQ2**	**ASQ3**	
Onesait Healthcare Data	Patients	1. You want to manually register a weight value/blood pressure.	6.50	6.50	7.00	6.67
		2. You want to access your CHF program to fill in a health questionnaire.	7.00	6.50	7.00	6.67
		3. You want to access your CHF follow-up program and consult your personal care plan.	7.00	6.50	7.00	6.67
	Caregivers	1. You want to manually register a weight/blood pressure value of Mrs. Z.	7.00	7.00	7.00	7.00
		2. You want to access to the follow-up details of Mrs. Z.	6.67	6.67	6.83	6.72
		3. You want to access Mrs. X´s CHF program to fill in a health questionnaire.	6.67	6.67	7.00	6.78
		4. You want to access Mrs. X´s CHF follow-up program and consult her personal care plan.	6.83	7.00	7.00	6.94
	Health Professionals	1. You want to access Mrs. X CHF follow-up program.	7.00	6.67	6.83	6.83
		2. You want to perform the initial clinical assessment to Mrs. X.	6.75	6.58	6.58	6.64
		3. You want to create and publish the personal care plan (PCP) for Mrs. X through a template so she can consult it through the MyHealth App.	6.58	6.50	6.50	6.53
		4. You want to customize what measures (vital signs) Mrs. X must include in the CHF follow-up.	6.92	6.83	6.92	6.89
		5. You want to include a weight range between 50 and 62 kg, outside of which you will receive an alert for Mrs. X.	6.83	6.58	6.58	6.67
		6. You want to customize what health questionnaires Mrs. X must fill in from MyHealth App and how she will have them available: only once, or anytime to be filled in whenever she wants.	6.42	6.83	6.42	6.56
		7. You want to consult the last records of Mrs. X’s heart rate and her heart rate history (Smartband).	5.92	6.25	6.08	6.08
		8. You want to consult the history of personalized follow-up plans of Mrs. X (the details of the plan, which professional published it and when).	6.92	6.92	6.92	6.92
		9. You want to know if Mrs. X has used the washing machine (or any other device that is being monitored) this week (MIW+).	6.60	6.50	6.40	6.50
		10. You want to check that you receive an alert when the alarm configuration is fulfilled (Amicare).	6.33	6.25	6.25	6.28
		11. You want to check what patients have alerts from the list of patients of the CHF follow-up program.	6.58	6.67	6.50	6.58
		12. You want to finalize the Mrs. X´s CHF follow-up program.	6.67	6.67	6.67	6.67

**Table 13:** SUS mean scores for Onesait Healthcare Data with the integrated technologies during the pre-validation phase 2.

Technology	Type of User	Mean Scores for Individual Questions										SUS Mean Score
		SUS1	SUS2	SUS3	SUS4	SUS5	SUS6	SUS7	SUS8	SUS9	SUS10	
Onesait Healthcare Data	Patients	4.38	1.50	4.75	1.75	4.75	1.50	3.88	1.38	4.38	2.00	85.00
	Caregivers	4.67	1.17	5.00	1.00	4.83	1.16	4.16	1.16	4.83	1.16	92.50
	Health Professionals	4.33	1.67	4.75	2.00	4.33	1.25	3.92	1.33	4.58	1.25	86.04

## Data Availability

All the data reported in this work are private, including the software developments, available in the private repository GitLab https://gitlab.com/pharaongroup (accessed on 29 September 2022).
